# The Müller-Lyer Illusion in Ant Foraging

**DOI:** 10.1371/journal.pone.0081714

**Published:** 2013-12-11

**Authors:** Tomoko Sakiyama, Yukio-Pegio Gunji

**Affiliations:** Department of Earth & Planetary Science, Faculty of Science, Kobe University, Nada, Kobe, Japan; University of Western Australia, Australia

## Abstract

The Müller-Lyer illusion is a classical geometric illusion in which the apparent (perceived) length of a line depends on whether the line terminates in an arrow tail or arrowhead. This effect may be caused by economic compensation for the gap between the physical stimulus and visual fields. Here, we show that the Müller-Lyer illusion can also be produced by the foraging patterns of garden ants (*Lasius niger*) and that the pattern obtained can be explained by a simple, asynchronously updated foraging ant model. Our results suggest that the geometric illusion may be a byproduct of the foraging process, in which local interactions underlying efficient exploitation can also give rise to global exploration, and that visual information processing in human could implement similar modulation between local efficient processing and widespread computation.

## Introduction

The Müller-Lyer illusion [1]–[3] has previously been explained by proposing that a stimulus with arrow tails signifies a concave corner in the 3D world, whereas a stimulus with arrowheads signifies a convex corner. Thus, the length of the central shaft, corresponding to the central edge of the two types of corners, is compensated by misapplied perspective [4], [5]. This “Top-down” explanation has been rejected because variations in which the arrowheads and tails are replaced by squares or dots can also give rise to the same illusion [6], [7]. However, the basic explanation remains that the discrepancy between a geometric stimulus in the 3D physical world and the projected 2D field can generate the Müller-Lyer illusion. In particular, Howe and Purves [8]–[10] have suggested that the anomalous perception of length is explained by the statistical relationship between the lengths of retinal projections and the lengths of their real-world sources. This relationship demonstrates that the 3D real world is analogously evaluated by processing of the 2D visual field. However, the problem still remains because a blind man who never experience statistical relationship between 2D visual cues and 3D resources can also recognize Müller-Lyer illusion [11].

According to several psychophysical studies, apparent line lengths are strongly biased by the neural-computation process of the weighted means of the stimulus distributions [12]–[15]. These are “Bottom-up” explanations. Thus, the anomalous perception can be explained by a neurophysiological model based on the finite resolution of visual-perception processing [16]–[22]. In addition to analogizing the 3D world by the 2D visual field, the finite computational resources must compensate for globally distributed patterns. In other words, the perceptional field, with its finite resolution, can form visual patterns for any optical stimulus. Thus, fine local structures within the image are received as erroneous, and uncertain patterns are filtered and altered by image processing with finite resolution. This phenomenon is called the uncertainty principle [17], [18]. In this sense, an economic balance is required to mimic a given visual pattern with relatively few local neural processors. There is a tradeoff between the economy of computational resources and the ability of producing a visual pattern apparently. The economic balancing of neural computation can generate the Müller-Lyer illusion as a byproduct.

Animals are also faced with an economic balance between global exploration and local exploitation [23]–[25]. However, diversity plays a role in balancing exploitation and exploration by disturbing the recruitment process [26]–[28]. For a swarm of ants, recruitment to a route connecting the nest with a food source can enable a colony to monopolize that food source. However, highly efficient exploitation, achieved by either pheromone trails or path integration, inhibits the colony's ability to discover new food sources through exploration [29]. This economic balance is analogous to the neurophysiological balance that generates the Müller-Lyer illusion.

If each ant in a colony can correspond to a neuron or retinal cell, then the behavior of a swarm of ants can correspond to the behavior of a neurological field. In this context, the Müller-Lyer illusion can be considered a particular pattern that can be produced by a foraging field of ants. This consideration means not that each ant might see an illusion but rather that a swarm of ants might see the Müller-Lyer illusion. Note that even an explanation that invokes a neurological field remains a problem of who sees a global pattern [30]–[32]. An observer who sees a global pattern as a whole is merely an assumption of visual processing models. Visual processing and illusion can be interpreted as a problem of pattern formation independent of an assumed observer.

Based on these observations, we designed an experiment to determine whether ant foraging patterns can generate the Müller-Lyer illusion. We provided honeydew distributed in the shape of the Müller-Lyer figure to garden ants (*Lasius niger*), which use visual information as a behavioral cue [33]. We investigated whether the resulting “ant field” produced the Müller-Lyer effect by extending or contracting the apparent length of the central shaft of the Müller-Lyer figure depending on the terminal-arrow directions. We then developed a foraging ant model to explain the Müller-Lyer effect.

## Materials and Methods

### Background for analogy

As mentioned before, top-down explanations for the Müller-Lyer illusion have still problems. The explanation based only on uncertainty principle is a typical bottom-up explanation referring no global property, which is proposed by the model based on spatial low pass filter [34], [35]. Carlson, however, showed that the illusion magnitude for the Müller-Lyer figure out of a stimulus which is free of low spatial frequencies is not significantly changed [36]. It suggests that bottom-up only model never explains Müller-Lyer illusion and that bottom-up model needs top-down feature such as global property of a pattern.

Human visual processing consists of two information processing, “what”-computing (identification of object) and “where”-computing (representation in a perspective). Because where-computing estimates various sizes and locations of a local pattern, and represents the pattern in a perspective, a local pattern is computed in referring to a global property of a visual scene. Recenty where-computing in inferotemporal cortex underlying visual object recognition is implemented by integration of two kinds of operation, a weighted linear summation and an elimination of the most frequent pattern (non-linear max operation) with respect to size and location, which is called HMAX model [19]–[22]. Since HMAX model is regarded as a coming model for visual object recognition, it is estimated whether Müller-Lyer illusion can be produced by HMAX model [15]. It is concluded that image-source statistics and reliance on information at low spatial frequencies are not necessary factors for generating the illusion, and that the Müller-Lyer illusion can be produced using only feed-forward, neurophysiological connections (i.e. non-linear max operation). The difference between the model and human, however, still remains. Although the illusion effect in human is perceived even for the Müller-Lyer figure with the wing angle 80 degree, it is no longer demonstrated for the figure with 60 degree in HMAX model.

Finally, both of top-down only explanations (misapplied size constancy scaling, the statistics of image-source relationships) and bottom-up only explanation (low pass filter) are collapsed, and we have to estimate bottom-up based model featuring global property like HMAX model. However, even HMAX model partly explains the Müller-Lyer illusion. We have to another idea for the device of featuring global property, and have to set up more general and simpler framework for pattern formation of the Müller-Lyer illusion because the HMAX model is too complicated to consider the basic mechanism.

**Figure 1:**
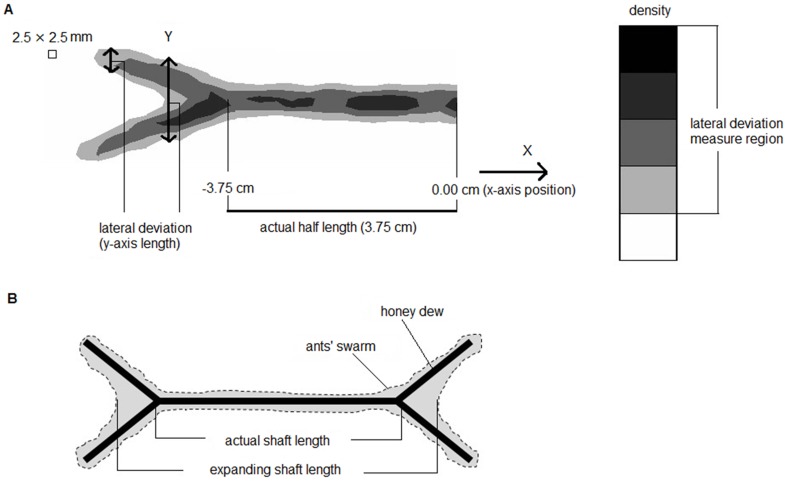
Schematic figures to explain the data analysis and evaluation of lateral deviation. (*a*). Sketch presenting half of a wings-out figure presented in Figure 2a and the density of black dots along the figure. The vertical line indicates a sample lateral deviation length. The numbers below the figure indicate the length along the shaft, with 0.00 at the center of the shaft and 3.75 cm at the wing branching point. The figure was divided into 2.5-mm segments. We restricted the measurement of measure lateral deviation length to regions with greater than 20% density. (*b*). Schematic figure explaining why the lateral deviation is attributed to the illusion effect. Black lines indicate honeydew. Gray zones indicate the boundaries of the ant swarm. The outward version i.e., expanding version, is illustrated here. The expansion (or compression, in the inward version) of the shaft length was determined by the maximum (minimum) position along the x-axis from the center (0.00 cm) that had a lateral deviation length of more than 1.0 cm.

In this context we compare the Müller-Lyer illusion in human to the foraging pattern of ants for the Müller-Lyer figure. The reason is the following. The visual information processing of human is employed to the retina, V1, vertical and ventral streams from V1 to the frontal region of the brain. The visual information processing thus can be implemented by a 2D model in which each cell is connected with near cells and far cells (e.g. HMAX model). First, we can see the analogy between them on a phenomenological level, because a cell in a visual system can be compared to an individual ant, and generated pattern for a visual stimulus can be compared to the distribution of ants for a feeding stimulus. In the preliminary experiment, it is found a particular blur pattern for a feeding stimulus in the Müller-Lyer figure as shown in Figure 1. Thus we can define apparent length of the diagram by the length of blurred pattern of ant distribution. This estimation for the apparent length for the generated pattern is as same as ones in various bottom-up visual models [15].

Second, we can see the analogy on mechanistic level. The HMAX model suggests the importance of integrating global property or long range interaction. However the long range interaction implemented by non-linear MAX operation cannot explain the Müller-Lyer illusion. In ant foraging, each ant can detect long ranged information via other ants and/or pheromones. If these kinds of indirect long-ranged information play a role in production of the illusion, it can give us a new knowledge on the implementation of the global property embedded in local interaction in a human visual system.

Third, there might be analogy even on a perception level. Most of the readers agree with the definition of illusion as a perception, as of visual stimuli that represents what is perceived in a way different from the way it is in reality. In this definition, one subject is asked to indicate the aspect of his perception. Therefore, one can feel the gap between human illusion and illusion pattern produced by ant. One unique subject perceives a visual illusion in human, while there is no one behavior for all individuals of ant swarm. If apparent length of the figure is defined by the length of a blurred pattern, one might find a cue of perception in the behavior of “some” ants concentrated in a marginal area of blurred pattern. Such a particular behavior is not propagated among all ants in a swarm, which could entail the gap. Some ants “perceive” an illusion, but others do not.

Libet [37], [38] discovered that even a consciousness of human is not unique. Although voluntary action is caused by unconscious readiness potential, intentional consciousness which is just a part of the brain interprets the cause of the action in the form of postidction (i.e. retrospective inference). It shows that some part of the brain controls action without consciousness and others has nothing to do with the action with consciousness [39]–[41]. Even a subjective consciousness consists of some parts which have no identical perception and could be an illusion [42], which can be compared to a swarm of ants without common perception.

In this perspective we first show the phenomenological analogy between the Müller-Lyer illusion and foraging pattern of ants. Given a feeding stimulus in the form of the Müller-Lyer figure, distributions of feeding ants are estimated and compared to the Müller-Lyer illusion. Second, we propose the ant foraging model which can show the Müller-Lyer illusion, and discuss the analogy on the mechanistic level, which might refer to the analogy on the perception level since even a subjective consciousness is not regarded as a unity.

### Rearing conditions

We studied two queen-less *Lasius niger* colonies with 300–500 workers. The colonies were collected at Kobe University and housed in plastic foraging boxes (35.1×25.5×6.1 cm), each of which contained a plastic nest box (5.1×5.5×1.1 cm) covered with clear red plastic sheets. The walls of the foraging boxes were coated with talcum powder to prevent the ants from escaping. The nests were regularly moistened, and the colonies were maintained at room temperature (26.1°C). All experiments were conducted in a room with artificial lighting. Fresh water was provided continuously. The colonies were fed twice per week with honeydew and once per week with mealworms. To ensure robust swarming behavior, the colonies were starved for five to seven days prior to each two-day experiment.

### Experimental setup

We brush-painted honeydew solution (50% w/w) on cardboard in the shape of the Müller-Lyer figure using templates cut from a transparent plastic sheet. The Müller-Lyer figure consisted of a 7.5-cm central shaft with two 3-cm wings pointing either inward (wings in, < – >) or outward (wings out, > – <). The line width was 2.5 mm. The wings were angled at 23.75° relative to the shaft for the wings-in configuration and 156.25° relative to the shaft for the wings-out configuration.

Previous studies have found that human Müller-Lyer errors increase when the wing angles of the stimulus are smaller [16], [43], [44]. Therefore, we performed large angled figure tests using figures with wings angled at 38.75° for the wings-in configuration and 141.25° for the wings-out configuration to determine whether sharper wing angles are associated with an increase in the number of foragers swarming to non-reward areas near the pivot, producing an expanded or compressed shaft (see below). Using a simulation model, we discuss why more foragers swarm to non-reward areas near the pivot when the wing angle is sharper.

The cardboard with the honeydew Müller-Lyer figure was installed in a plastic foraging box, and the ant swarming to the figure was recorded with a video camera (Sony). For each colony, one trial lasting approximately 10–20 min was conducted per day. Two trials were conducted for each version of the figure (i.e., wings-out, wings-in, large angled wings-out, large angled wings-in).

We used image-processing software (ImageJ; Rasband, W.S., National Institutes of Health, Bethesda, Maryland, USA) to track the ants' trajectories. In our experimental analysis, ants were converted to black dots, and the background was converted to white space. Then, we obtained x–y coordinates for each of the black dots by assigning each 2.5 mm of actual grid size i.e. approximately individual forager body size to a single pixel (eight frames per second). The density of the black dots was then calculated using the Gaussian kernel density estimation function (bandwidth set to (1,1)) in R version 2.14.0. To eliminate the excessive black dot densities on the actual honeydew-painted regions, x–y values that did not change from frame to frame were excluded from the analysis.

## Results

### Experimental results

**Figure 2:**
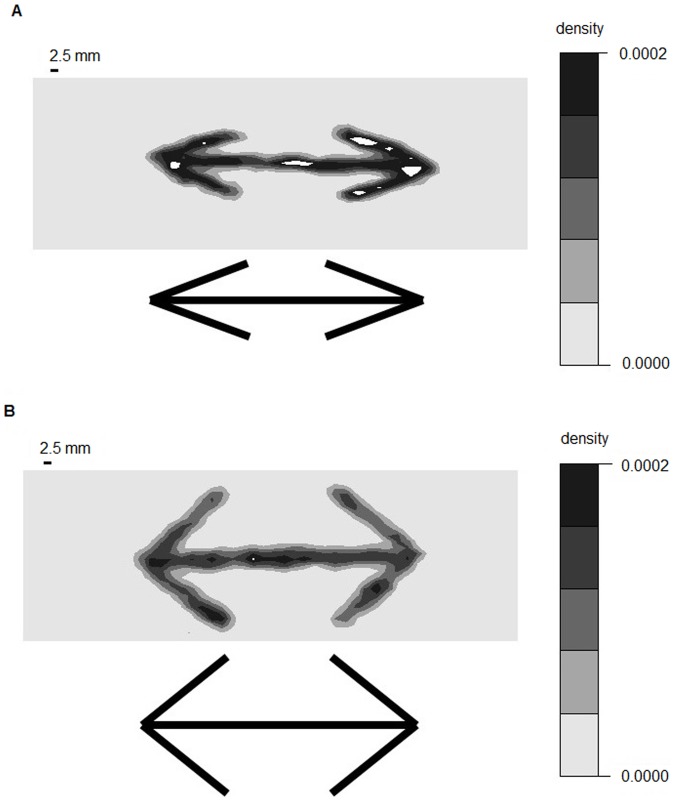
Examples of black dot density in outward and large angled -outward experiments. (*a*). Wings-out configuration. (*b*). Large angled wings-out configuration. White areas were excluded from analysis due to excessive ant density.

**Figure 3:**
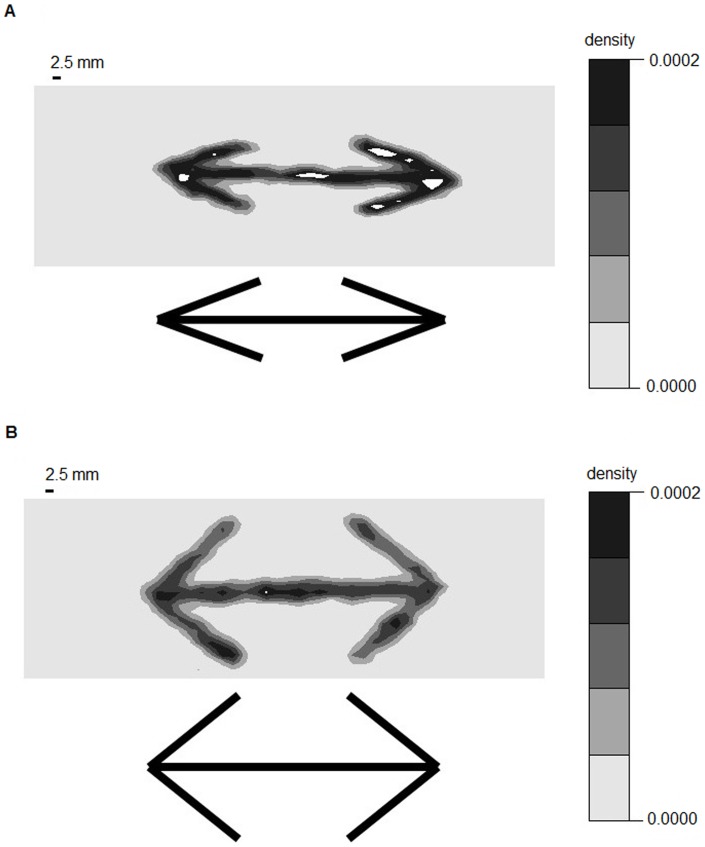
Examples of black dot density in inward and large angled -inward experiments. (*a*). Wings-in configuration. (*b*). Large angled wings-in configuration. White areas were excluded from analysis due to excessive ant density.

To analyze the Müller-Lyer effect, we divided the length of the figure along the x-axis into 2.5-mm segments from its maximum values (6.25 or −6.25) and measured the lateral deviation in each segment (Figure 1a). Lateral deviation was defined as the length of the black dot-dense regions (more than 20% density) along the y-axis at each position along the x-axis, which was computed as shown in Figures 2 and 3. Note that a slight amount difference of honey dew solution caused to overall ant density different, even though our extreme care of that. The lateral deviations were considered to contribute to the formation of formation of outer closed or inner closed wings, resulting in an expanded or compressed shaft (See Figure 1b). Then, the illusion effect i.e., shaft expanding/compressing position along the x-axis, was assigned to the position of the lateral deviations. If ant density between the two arrow tails was lower than 20%, the lateral deviation on that x-axis were calculated from one of two tails which had longer lateral deviation than the other only restricting to >20% density regions (See Figure 1a). We set the maximum (minimum) x-axis position that had more than 1.0 cm lateral deviation length as the expanding (compressing) position from the center shaft position (0.00 mm) and obtained the effect percentage defined as (expanding – compressing)/baseline.

We calculated the mean effect percentage by dividing the figure in half because it is not necessarily secure to be perceived these divided half figures as same ones and due to the asymmetric distributions of the black dots, which could extend further beyond the underlying honeydew shaft on one side than on the other. The baseline refers to the actual half-length of the actual painted honeydew shaft (3.75 cm). Thus, we obtained two effect percentages from each outward and inward trial pair.

**Figure 4:**
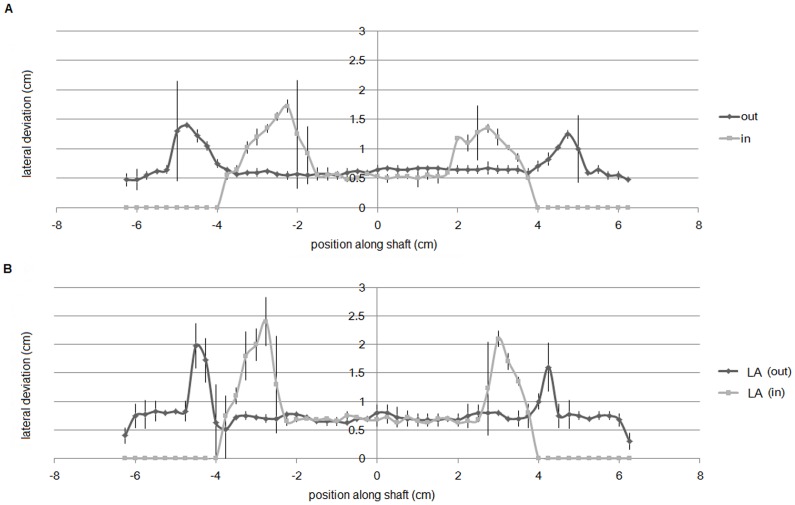
The relationship between lateral deviation and position along the shaft. (*a*). Wings-out and wings-in configurations; “out” and “in” indicate wings-out and wings-in, respectively. (*b*). Large angled configurations; “LA (out)” and “LA (in)” indicate wings-out and wings-in large angled configurations, respectively. Two trials were conducted for each of the four configurations (outward, inward, large angled -outward, large angled -inward), and the average values were plotted. Lateral lines indicate error bars.

**Figure 5:**
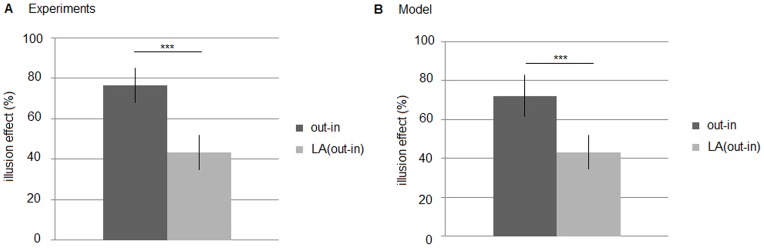
Mean effect percentage of the Müller-Lyer illusion. (*a*). Experimental results. (*b*). Model results. “Out-in” indicates the wings-out and wings-in configurations; “LA (out-in)” indicates the large angled configurations (***P<0.005). Lateral lines indicate error bars.

Figure 4 presents the lateral deviations plotted against every 2.5-mm position. The wings-out (> – <) figures appear to show longer shaft lengths than the wings-in (< – >) figures. This tendency is stronger in the figures with smaller wing angles. Figure 5a presents the mean effect percentages of the two versions of the figures (wings-out/wings-in vs. large angled wings-out/wings-in). The high effect percentage of the wings-out/wings-in figures indicates that the differences in the distances between the expanding and compressing length is larger for these figures than for the large angled figures (Welch two-sample t test: *N* = 4 vs. 4, *t* = 5.48, *df* = 6, *P*<0.005).

**Figure 6:**
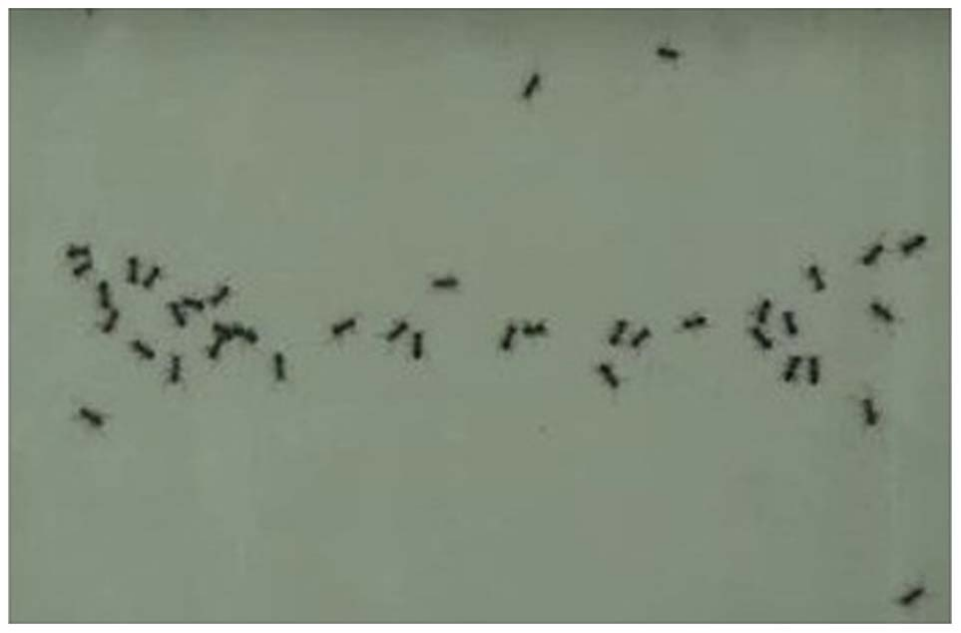
A representative snapshot illustrating a distribution of ants. The wings-out configuration is shown.

Figures 2, 3, and 6 provide examples of the experimental tests, which demonstrated that the ant density was greater in wing areas than in areas without food and that angle sharpness contributed to this pattern.

**Figure 7:**
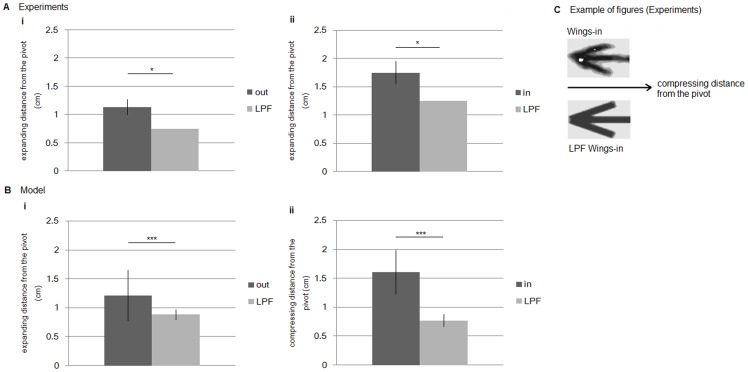
Average expanded (compressed) positions from the experimental results (actual ant concentration) and low-pass filtered results. (***a***). Experimental results. “Out” (“In”) indicates experimental Wings-out (Wings-in) results; “LPF” indicates low-pass-filtered effect results. (*b*) Model results. (*c*). Schematic figures of compressed positions (Wings-in) of experimental results. Lateral lines indicate error bars. *P<0.05.

We also investigated whether the Müller-Lyer illusion generated by ants might be explained by simple low-pass filtering effects. Even in this case, the blur of a given diagram line thickness and the location of the junction of the lines are shifted; junctions are shifted inwardly in arrowheads (toward the central point of the shaft), and they are shifted outwardly and in arrow tails. This effect could also explain the change in the shaft length, whereas the distance of the shift resulting from the low low-pass filter is expected to be smaller than the distance of the shift resulting from foraging ants Figure 7c). If so, the blur would be expected to occur with equal probability anywhere in the diagram.

We define the blur effect due to the low-pass filter for the Müller-Lyer illusion as follows: Given an accumulated pattern of ant distribution in the experiment, we first obtain apparent effect of the blur for a honeydew line by measuring the width of the thickened line. The width of the thickened line was calculated as the average shaft width of following positions along the x-axis (0.00, ±0.25, ±0.50, ±0.75 cm). Figures 7a illustrates that the actual ant concentration represents significantly expanded shaft lengths from the pivot (Wings-out: Mann Whitney U test, *N* = 4 vs. 4, *U* = 0.00, *P*<0.05, Wings-in: Welch two-sample t test: *N* = 4 vs. 4, *t* = 2.78, *df* = 5.85, *P*<0.05, Note that we used two different static analyses depending on whether the data showed normality or not).). This observation indicates that our results are unlikely to be due to simple low-pass filtering effects.

### Foraging ant model generating the Müller-Lyer illusion

**Figure 8:**
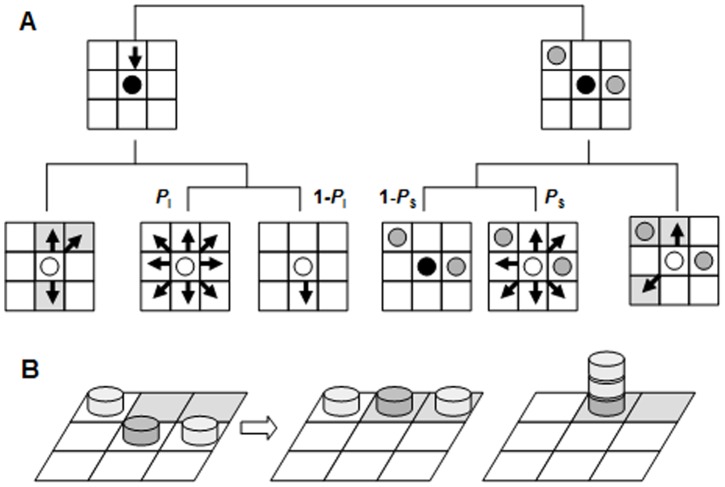
Schematic diagram of the foraging ant model. (*a*). Movement rules of the foraging ant model. An ant at the center of a neighborhood (black circle) chooses its next location depending upon the conditions of its neighborhood. The first condition is the presence or absence of other ants, depicted as gray circles (top row). The black arrow represents the ant's direction of movement. The second condition is the presence or absence of a food source, depicted as gray cells. Finally, the probabilities of different possible movements are determined by *P*_I_ and *P*_S_. The possible movements are represented by black arrows (bottom row). (*b*) Difference between asynchronous (center) and synchronous (right) updating in the next state resulting from a given previous state (left). Gray coins represent foraging ants.

We here propose a foraging ant model to explain the Müller-Lyer effect. This model is based on an ant agent walking in a grid space according to specific rules (Figure 8a). The grid space consists of 90×90 cells with edge wrapping. Each cell is either a blank cell or a food source. An ant can remain in any cell, with one ant per cell when the transition rule is applied to each ant. Each ant sees only the eight nearest neighboring cells, called the neighborhood. If there is no other ant in the neighborhood and one or more food sources are available, an ant moves to one of the food sources at random. If no food source is available in the neighborhood, the ant continues to follow its previous direction of movement with probability 1-*P*_I_ and changes its direction of movement with probability *P*_I_. If there are other ants in the neighborhood and one or more food sources are present, the ant moves to an unoccupied food source. If no food source is available, the ant stops with probability 1-*P*_S_; otherwise, it moves to a randomly selected blank cell with probability *P*_S_ (Figure 8a).

The model can operate either synchronously or asynchronously. Under synchronous updating, each ant simultaneously follows the rules of movement defined in Figure 8a. Under asynchronous updating, the ants move in an order that is randomly determined at each time step. Figure 8b illustrates the differences between asynchronous and synchronous updating. If three ants are located as shown in Figure 8b (left), asynchronous updating permits two of them to move to the two food sources and avoid overlap. In contrast, synchronous updating can result in three ants overlapping at one food source, as shown in Figure 8b (right). The synchronous updating disturbs the principle of one ant per cell. Thus, asynchronous updating can generate efficient exploration of scattered food sources.

This model has only two parameters, *P*_I_ and *P*_S_. Smaller values of *P*_I_ correspond to less frequent changes in the direction of movement. Smaller values of *P*_S_ correspond to more frequent stopping near other ants. If the given space is relatively small, even small values of *P*_I_ can enable the ants to explore the entire space. When *P*_I_ is small, small *P*_S_ values enable the ants to achieve efficient exploitation. Because ants that stop at a food source do not move, ants that remain close to other ants can approach the food source even if they do not reach it directly. Thus, small *P*_S_ values cause ants to remain in the area of a food source for a longer period, resulting in efficient exploitation. In all simulations, parameter values of *P*_I_ = 0.05 and *P*_S_ = 0.1 were chosen and 300 ants were simulated.

**Figure 9:**
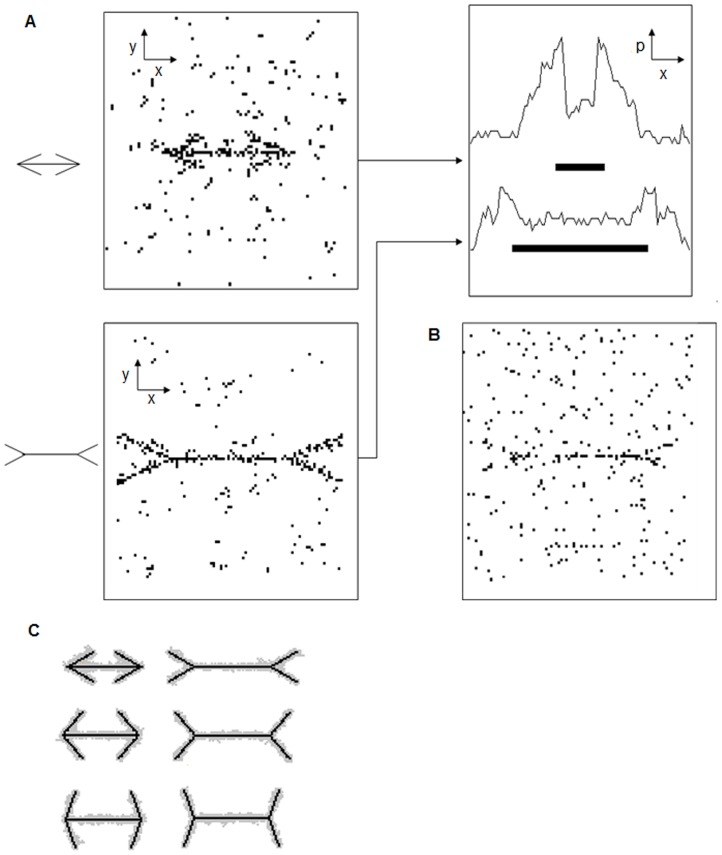
Results of the foraging ant simulation model. (*a*). Steady-state patterns generated by 300 ants initially distributed randomly with food sources distributed along the wings-in (upper left) and wings-out (lower left) Müller-Lyer figures and peak length plotted against the position along the x-axis (right). The movement rules described in Figure 6 are operated asynchronously. (*b*) Steady-state patterns under the same conditions as (*a*) except with synchronous updating. (*c*). Accumulated patterns over 6000 time steps, for various Müller-Lyer figures. A gray cell represents the cell with high density exceeding 300 ants per cell, and black represents the food source.

In each simulation, food sources were distributed along the wings-in (< – >) or wings-out (> – <) Müller-Lyer figure, and 300 ants were initially scattered randomly on the grid. After more than 100 time steps, the ants were distributed on the Müller-Lyer figure (Figure 9a left). Although steady-state patterns were obtained, the arrowheads or tails were clearly connected to the central shaft. More ants were clearly concentrated in a narrow local area intersected by the arrow branches in both the wings-in and wings-out configurations. To estimate the apparent length of the central shaft (i.e., the expanded or compressed shaft length), the lateral deviation defined in Figure 1a was calculated for the cumulative pattern generated by 10,000 time steps. Figure 9c shows a distribution of accumulated patterns over 2000 time steps. The length of lateral deviation along the y-axis plotted against the x-axes for the wings-in and wings-out configurations is shown in Figure 9a (right). In simulation model, the apparent length of the central shaft of the Müller-Lyer figure is defined by the distance between the maximum (minimum) position along the x-axis from the center on the right and left sides in the outward (inward) version. The apparent length of the shaft is clearly smaller in the wings-in configuration than in the wings-out configuration. Figure 9b presents the steady-state pattern generated by the ant model with synchronous updating. Overlapping in particular cells prevents the ants from concentrating exclusively on the food sources throughout the grid, inhibiting the conspicuous formation of the Müller-Lyer figure.

**Figure 10:**
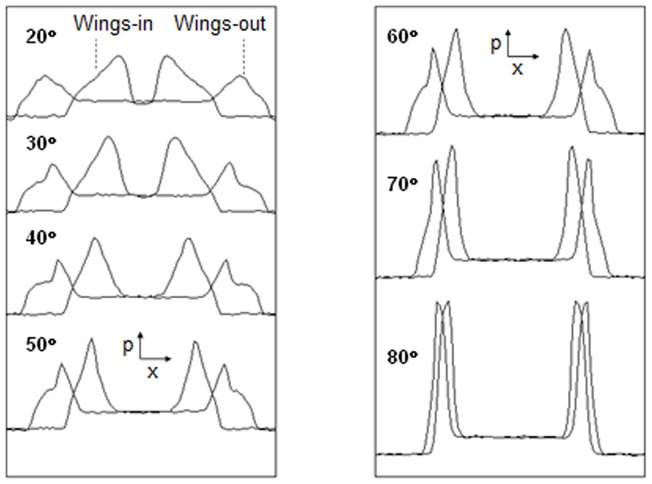
Peak length plotted against position along the x-axis for various wing angles.

**Figure 11:**
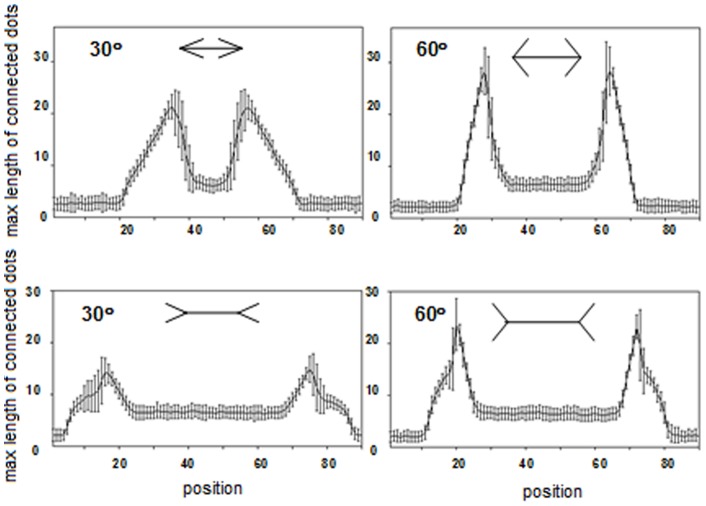
Application of lateral deviation versus position along the x-axis for various wing angles to calculate the mean effect of the Müller-Lyer illusion. The curve represents the mean value over 100 samples. Each sample represents 10,000 cumulative snapshots of simulations. The vertical bars along the curve represent the standard deviation.

In Figure 10, the lateral deviation of the wing portion with various wing angles is plotted against the position along the central shaft for a pair of wings-in and wings-out Müller-Lyer figures. The angle intersected by the branches (arrowhead or tail) is equal for the wings-in and wings-out figures of each pair. Each graph represents the mean value of 100 trials similar to that shown in Figure 9. The apparent length of the central shaft of the Müller-Lyer figure is defined by the distance between the x values with the greatest lateral deviation. Thus, the illusion effect is expressed as the difference between the apparent lengths of the wings-in and wings-out configurations. Larger angles produce smaller illusion effects. This tendency is consistent not only with the results of our ant foraging experiment but also with the human perception of the Müller-Lyer illusion [16], [43], [44]. To compare the simulation results to the experimental results for *L. niger*, we focused on the Müller-Lyer effect for patterns with wide (60°) and narrow (30°) wing angles, as shown in Figure 11. The mean effect percentage was much greater for the narrow wing angle than for the wide wing angle (Mann-Whitney U test: *N* = 100 vs. 100, *U* = 9,858.5, *P* = 2.2e^−16^<0.05).

**Figure 12:**
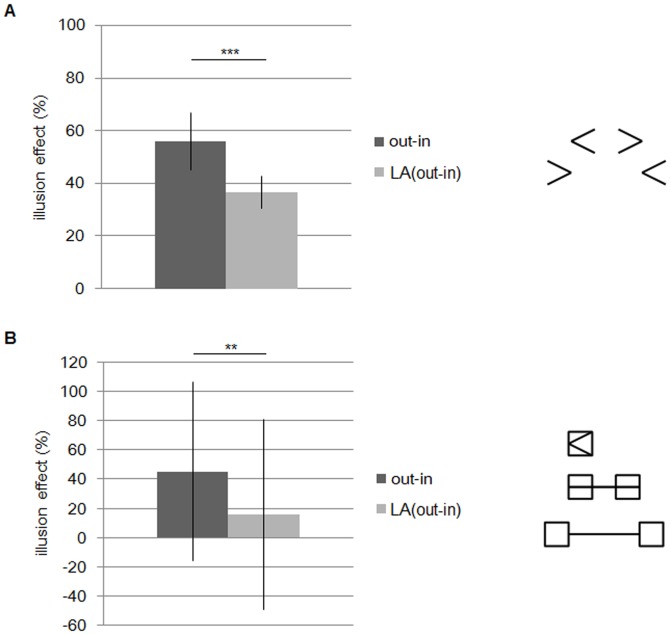
(a) The illusion effect in the ant foraging model for the variant of Müller-Lyer figures (wings-out/wings-in vs. large angled wings-out/wings-in) where central shaft is removed. (b) The illusion effect in the ant foraging model for the variants of the Müller-Lyer figure, where arrow head or tail is replaced by a box.

As mentioned before, top-down explanations by Gregory is rejected by the fact of which the illusion magnitude is not changed by replacing arrow head with box or by removing the shaft [7]. We here show that our ant model also produces the illusion effect for those variants of the Müller-Lyer figure. Figure 12a presents the mean effect in the ant foraging model for the variants of the figures (wings-out/wings-in vs. large angled wings-out/wings-in) where the central shaft is removed. The variant of the Müller-Lyer figures without central shaft are shown in the right of Figure 12a. As same as Figure 5, the wing is intersected by the shaft with 23.75° for the wings-in and 156.25° for the wings-out configuration. Large angled figure is with wings angled at 38.75° for the wings-in and 141.25° for the wings-out. The high effect of the wings-out/wings-in figures indicates that the differences in the distances between the expanding and compressing length is larger for these figures than for the large angled figures (Mann-Whitney U test: *N* = 40 vs. 40, *U* = 122.0, *P* = 4.75e^−11^<0.01). Figure 12b shows the illusion effect for the variants of the Müller-Lyer figure produced by the ant foraging model, where a wing is replaced by a box introduced by the wing is intersected by the shaft with 23.75° for the wings-in and 156.25° for the wings-out (Figure 12b right). The large angled figure experiment represents the pair of the figures with vertically extended boxes introduced by the shaft with 38.75° for the wings-in and 141.25° for the wings-out. The larger the angle introducing the box is, the higher the effect is. Two effect is significantly different (Mann-Whitney U test: *N* = 40 vs. 40, *U* = 526.0, *P* = 0.0083<0.01). These results show that our ant foraging model also produces the Müller-Lyer illusion even for the deformed figure, as well as human perception.

Finally, we compare the simulating results with the ant experiment. Figure 5b shows the mean effect produced by the ant foraging model for the two versions of the Müller-Lyer figures (wings-out/wings-in vs. large angled wings-out/wings-in) which are the same as those used for the ant experiment in Figure 5a. The line width is compared to one cell in a two dimensional model, and the shaft and arrow length are given in the same proportion as the figure in the real ant experiment. The wing angles intersected by the shaft are the same as those of the ant experiment (23.75° for the wings-in and 156.25° for the wings-out, and in the large angled figure 38.75° for the wings-in and 141.25° for the wings-out). As well as the results of the ant experiment, the illusion effect of the 23.75° and 156.25° pair is larger than that of the 38.75° and 141.25° pair (large angled), and the difference is significant (Mann-Whitney U test: *N* = 40 vs. 40, *U* = 60.0, *P* = 8.01e^−13^<0.01). Compared with the illusion effect in the ant experiment with one produced by the ant foraging model, the magnitude of the illusion effect are almost same, about 75% for the 23.75° and 156.25° pair, and about 40% for the large angled pair. Actually, there were no significant differences of mean effects between experiments and models in both versions (wings-out/wings-in(experiment) vs. wings-out/wings-in(model): Mann-Whitney U test: *N* = 4 vs. 40, *U* = 57.0, *P* = 0.35, NS, large angled wings-out/wings-in(experiment) vs. large angled wings-out/wings-in(model): Mann-Whitney U test: *N* = 4 vs. 40, *U* = 73.0, *P* = 0.78, NS). However, there were significant differences between different version's experiment and model (wings-out/wings-in(experiment) vs. large angled wings-out/wings-in(model): Mann-Whitney U test: *N* = 4 vs. 40, *U* = 2.0, *P* = 0.0011<0.01, large angled wings-out/wings-in(experiment) vs. wings-out/wings-in(model): Mann-Whitney U test: *N* = 4 vs. 40, *U* = 0.0, *P* = 0.0011<0.01). These results indicate that the foraging model can explain the Müller-Lyer illusion generated in the ant experiment.

The illusion effect due to the low pass filter is also estimated for the pattern produced by the ant foraging model. As well as the estimation for the pattern generated in the ant experiment, the width of the blur was calculated as the average arrow width along the shaft. Figure 9c shows blur (gray area) distributed along a given Müller-Lyer figure. The condition for the steady state is the same as that of Figure 5b. The expansion of the ant concentration is defined by the length from the pivot of the Müller-Lyer figure to the margin of the blur in steady state along the shaft. Figures 7b illustrates that the ant concentration in the model represents significantly expanded shaft lengths from the pivot over the blur for both Wings-out with156.25° and Wings-in with 23.75° (Wings-out: Mann Whitney U test, *N* = 20 vs. 20, *U* = 58.00, *P* = 0.00011<0.05, Wings-in: Mann Whitney U test, *N* = 20 vs. 20, *U* = 0.00, *P* = 5.88 e^−8^<0.01). This observation indicates that the Müller-Lyer illusion produced by the foraging model is unlikely to be due to simple low-pass filtering effects. The values of the expanding distance over the blurred area in the foraging model are very similar with those obtained in the actual ants experiment. Through the comparison in Figure 5 and 7, it is verified that the foraging ant model is consistent with the ant experiment.

## Discussion

While the Müller-Lyer illusion could not explain either by top-down or bottom-up approaches, it can be produced by a visual processing model based on neurophysiological connections featuring feed forward, which is bottom-up system based on local (short ranged) interaction embedding longer ranged information. The problem how local neurophysiological interaction embeds longer ranged information processing still remains because the feed forward neurophysiological connection cannot explain the illusion effect remaining for Müller-Lyer figure with the large angles of arrow heads [15].

We here consider this problem can be helped by other more general approaches. As analogously as a local neurophysiological connection could refer to global property or longer ranged information, foraging animal which individually explores a limited local area can refer to other far area by using various information devices. Especially social animals can utilize information carried by other animals, which can compensate individual's limited ability on exploration [24], [29]. In our model it is assumed that an ant stops moving if it finds other ants in its own neighborhood, and that the rule is applied to each ant in asynchronous updating. While the former rule partly contributes to production of long range information, the effect is restricted and it also entails the overflow of information (i.e. overlap of ants at a single site). The asynchronous updating can prevent an ant from overlap at a single site, and can implement much longer range information propagation due to the chain reaction. In synchronous updating only an ant close to the feeding ant can stop at each time step. In asynchronous updating the ant can stop close to the ant close to the feeding ant due to the time lag. This effect can make an ant far from the food can be attracted to the feeder, and can lead to the chain reaction of the effect.

In this sense we first estimate whether the distribution of foraging ants can produce the Müller-Lyer illusion. When the apparent length of the shaft is defined by the length between blurred areas of ants distribution, the distribution of foraging ants could explain that the apparent length in the wings-out (> – <) pattern were longer than that of wings-in (< – >) pattern, and that the more illusion effect is the smaller the angle intersected by the wing and central shaft is. Because these properties are essential feature in the Müller-Lyer illusion in human, we can conclude that there is an analogy between the Müller-Lyer illusion in human perception and that in ant foraging on phenomenological level. Our results on the ant experiment clearly demonstrate that *L. niger* can produce the Müller-Lyer illusion.

In addition, studies in humans have found that blind people perceive the same illusion haptically rather than visually [11]. Furthermore, the shaft portion of the figure is not necessary to produce the illusion [6], [7]. Therefore, the Müller-Lyer illusion can occur without the perception of the entire figure and with non-standard versions, such as shaft-less figures. Since these findings support that the illusion can be basically local or bottom up process, it can also support our experimental results, because each individual ant cannot perceive a whole figure and the distribution of foraging ants is basically bottom up fashioned.

We next estimate the analogy between not only on phenomenological level but on mechanistic level, and we propose the ant foraging model based on local interaction and asynchronous updating. Then we compare the results of the ant experiment and our foraging model with respect to the illusion effect and the effect of low pass filter. It is found that although both of illusion is produced due to the blur of the distribution of concentrated ants, the blurred effect is specific to the narrow area intersected by wings and central shaft beyond the effect of low pass filter. In addition the effect of the illusion of the model is as same as that of the ant experiment. The model also shows that the more illusion effect is, the smaller the angle intersected by arrowheads and central shaft is. In addition, the model can show the illusion effect for the shaftless figure and the figure of which the arrow heads are replaced by boxes. These results suggest that the ant foraging is analogous to the visual processing not only in phenomenological level but in mechanistic level.

In our foraging ant model, each ant can perceive only its own neighborhood. Although its foraging ability is thus local and limited, the rule of which an ant stops if other ants is found in the neighborhood can help foraging behavior. Because an ant in the model always goes straight and stopped ant can move again randomly with some probability, other feeding ants which are found in the neighborhood can help the ant to approach the food sources. In our model, food sources are distributed linearly and locally, in the form of the Müller-Lyer figure. Thus, ants stopping at food sources distributed at the Müller-Lyer figure will attract other ants to be stopped near the food source. Because this attraction is feed-forward process, the concentrated ant area is blurred along the Müller-Lyer figure. The rule that ants must remain at sites near other ants can enable the ants to efficiently explore the area and to find out food sources, and exploit the food source (i.e., by feeding for a long time).

Although an ant's vision is limited to its local neighborhood, ants can obtain global information from other ants. In our model, the rule that ants must remain close to other ants may represent the use of information carried by other ants because it may help other ants to find food sources in the neighborhood. Real ants can use trail pheromones to efficiently utilize information carried by other ants. Our model suggests that ants can make use of global information even if they rely on the visual locations of other ants rather than on trail pheromones. This property is modulating the dilemma of exploration and exploitation, which can contribute to the production of the Müller-Lyer illusion.

The most important device is asynchronous updating in the foraging model. Asynchronous updating generates more efficient exploration than does synchronous updating. If the ant positions are updated synchronously, then multiple ants can become concentrated on the same site. Thus, the Müller-Lyer pattern is not fully produced in the ant foraging field. In other words, vision never holds. In contrast, asynchronous updating produces the full Müller-Lyer pattern because the ants can distribute themselves over the entire space without overlapping. Asynchronous updating can balance exploration and exploitation in this foraging ant model, thus generating the Müller-Lyer illusion. This result indicates that balancing exploration and exploitation can yield exploration with finite resolution, which contributes to the “uncertainty principle”. This process is analogous to the neurophysiological model, in which uncertain and erroneous patterns intersected by lines are filtered and altered, producing the Müller-Lyer effect. Our results suggest that any biological computation that balances exploitation and exploration at a finite resolution can produce anomalous perceptions, such as the Müller-Lyer illusion.

Conversely, our foraging model suggests that the asynchronous updating equipped with modulation could play an important role in human visual processing system underlying object recognition. The models of neural network connections pay little attention to the asynchronous updating, while actual neurons have a refractory period with randomly distributed deviation which can implement asynchronous updating. As well as ant foraging model, if neurons could inhibit or avoid too much feed-forward mechanism such as avoiding overlapping in the foraging model (i.e. modulation), surplus concentration of firing neurons at particular site can be avoided. The asynchronous updating with modulation can thus balance locally feed-forward acceleration with globally widespread distribution of firing neurons. It can give rise to economic and efficient visual system consisting of small amount of cells, which can implement resolution fine enough to discriminate objects in a visual scene, and can produce the Müller-Lyer illusion as byproduct.
